# Maize productivity amidst northern rural growth credit programme in Ghana

**DOI:** 10.1016/j.heliyon.2022.e10420

**Published:** 2022-08-28

**Authors:** Mutaka Mohammed Seidu, Mohammed Tanko

**Affiliations:** a Kwame Nkrumah University of Science and Technology, Department of Economics, Kumasi, Ghana; b University for Development Studies, School of Applied Economics and Management Sciences, Tamale, Ghana

**Keywords:** Agriculture, Credit, Government programme, Maize productivity

## Abstract

The need to improve maize production and develop agriculture led to the design and implementation of many flagship programmes in Ghana. Among these programmes is the rural growth credit programme. This paper used current data (2021) from credit-constrained maize farmers in the rural growth credit programme to extend the propensity score matching method to the analysis of credit impacts on farm productivity. The study used a sample of 130 farmers, comprising 65 farmers as a treatment group and 65 farmers as a control group. The findings of this paper indicate that, credit-constrained farmers who have access to the rural growth credit relatively have more productivity than credit-constrained farmers who did not have access to the credit. The paper therefore conclude that, the rural growth credit intervention program did achieved its intended purpose in respect of improving farm productivity in Ghana. It could therefore be deduced that credit interventions programs do have a positive impact on farm productivity in Ghana.

## 1 Introduction

Agriculture contributed 35% to Ghana's GDP between 2010 to 2015 (Bank of Ghana, 2018) and contributed significantly to job creation; 36% of the total labour force (3.3 million people) out of 9.3 million people formally employed are directly and indirectly engaged in the agricultural sector (Ghana Statistical Service, 2016). Agriculture in Ghana is largely informal, engaging 70.6% of the rural folk and contribute about 45% to foreign exchange though the average growth rate of the sector is estimated at 3.5% per annum between 2010 to 2016 (Bank of Ghana, 2018).

Credit-constrained impacts negatively on farmers’ welfare and income (Amanullah et al. 2020) but Abdulai et al., 2008, Abdulai and Binder, 2006, Abebaw and Haile, 2013, noted that, short term credit do not have any significant impact on farmers’ productivity. Eventhough there is a positive correlation between farm credit and farm investments (Carter, 1989; Foltz, 2004; Fletschner et al., 2010), a positive correlation between farm credit and farm productivity (Guirkinger and Boucher, 2008) and between farm credit and farm output (Feder et al., 1990; Akudugu, 2014, Akudugu, 2016, AmanullahLakhan et al., 2020, Awotide et al., 2015) but for Kochar (1997) there is no correlation between farm credit and farm efficiency. Despite these pool of research, the World Bank reports that Ghana is among developing countries with the lowest farm productivity amidst several farm credit modalities rolled out to boost the agricultural sector (Bank of Ghana Report, 2018).

According to Mugumaarhahama, Y et al. (2021) microfinace institutions often lend farm credit to only high income earners because of minimal risk of loan default, however these high income earners are often financially self sufficient (unconstraint credit farmers) and so the marginal impact of credit on their output may be insignificant. Past studies failed to distinguish between credit-constrained and unconstraint farmers when modelling the relationship between credit accessibility and farmers output (Hananu et al., 2015, Iddrisu et al., 2018, Imbens and Wooldridge, 2009, Johnson et al., 2011, Kassie et al., 2008). However, Sekyi, et al., 2017, noted that, modelling credit access and credit-constrained separately have a risk of yielding biased estimates and therefore a critical evaluation of the findings of these past studies revealed that farm productivity and farm credit may not be significantly correlated due to disparity in liquidly constrained and liquidity unconstrained farmers (Abdallah, 2016a, Abdallah, 2016b). The impact of credit on farm output will differ among farmers because of the variance in their credit needs. . This is to say that the additional contribution of farm credit to farm output will differ for farmers in the situation where credit is a binding constraint on their output functions. For farmers who are sufficiently self-financed, credit is not a critical constraint on their productivity functions. The farm credit aims to bring the farm input mix at or near the optimum point to yield the desired output level per farm acreage. When credit is a necessary constraint, the input mix will differ from optimal levels so that output levels may deviate from their optimal path.

On the other hand, if credit is not a necessary constraint, the input mix may still be optimal, and credit accessibility may have little or no impact on output levels. In this regard, to determine the real impact credit has on productivity, the study took note of sample selection bias between farmers who are credit-constrained and those who are not credit-constrained. This paper therefore, focuses only on credit-constraint farmers. To determined credit-constrained farmers, the study considered the World Bank poverty threshold of daily consumption of $1.9 per person of farmers househould and dependants. Farmers with income above this thresh hold were not considered for the study because they were considered as financially self sufficient (unconstraint credit farmers).

Empirical literature and policy documents always capture access to credit as one of the most important determinants of successful agricultural production in Ghana and elsewhere in the developing world, particularly in Africa (Barrett, 2001, Blundell and Smith, 1989, Di Falco and Bulte, 2011, Foltz, 2004b, Giang et al., 2015, Kochar, 1995, Martey et al., 2015, Minten, 2002, MoFA, 2013, Hammamet,). Agri-Economist and other experts argue that access to credit enables farmers to confidently invest in obtaining optimal input mix to ensure efficient yield levels (Akram, W., Hussaun, Z., Sail, M.H. and Hussain, J., 2008), which leads to poverty alleviation among farmers. However in the case of Natajan, Net al (2021), adverse incorporation resulting from debt bondage due to unfair lending practices among rural farmers can result in uneding poverty and adversely affect farmers productivity.

Theoretically, access to credit and farm productivity generally have a positive relationship. According to Dadson et al. (2014), individuals deposit their funds in financial institutions for safekeeping (financial institutions serve us delegated monitors for net-savers) and trust the financial institutions to invest these funds to yield returns for the depositors properly. Therefore, financial institutions are mandated to make sound judgments based on sufficient information and ensure that farmers who accessed credit used the credit judiciously to guarantee increased productivity to enable the farmers to pay back. According to the rational choice theory, individual farmers make the best choice of selecting credit models that best suit their circumstances and has the greatest possibility of translating into higher productivity.

The extent to which this long-held view being true in Ghana is uncertain because despite tremendous efforts made by the Government of Ghana and other stakeholders in advancing credit to farmers, World Bank still pegged Ghana's agricultural productivity among the lowest in sub-Saharan Africa (Di Falco et al., 2010, Doss and Morris, 2001, El-Shater et al., 2016, FAPDA and Foltz, 2015, Fuglie et al., 1995). Therefore, this paper aims at investigating the impact of farm credit on-farm productivity among credit-constrained farmers in Ghana using propensity scores to examine the average treatment among farmers who had credit against those who had no credit.

### 1.1 Style facts of Northern Rural Growth Programme intervention

The Northern Rural Growth Programme (NRGP) of the Ministry of Food and Agriculture (MOFA) is a Government of Ghana (GoG)/International Fund for Agricultural Development (IFAD)/African Development Bank (AfDB) initiative, with an overall goal of contributing to equitable and sustainable poverty reduction and food security among rural households on a sustainable basis in all districts of Northern Ghana, as well as seven adjoining districts of the Brong-Ahafo Region. Later, some districts in the southern part of the country were added specifically to finance the completion of schemes under the defunct Inland Valley Rice Development Project (IVRDP) and Small-Scale Irrigation Development Project (SSIDP) (Ayamga and Dzanku, 2013, Binswanger and Khandker, 1995, Dehejia and Wahba, 2002).

The survey and review of the program revealed the supported establishment of a total of 8,127 Farmer-Based Organizations (FBOs), exceeding the appraisal target of 2,000 FBOs by about 406 percent. This number of FBOs had a total membership of 201,746 people comprising 82,920 (41%) men and 118,826 (59%) women as direct programme beneficiaries. More than 50% of the FBOs were formed in just 2 years (2015–2016).

The NRGP has supported farmers in diverse ways and areas: in infrastructural development, including feeder roads and warehouse developments; managing post-harvest losses, improving farmers' access to extension services, irrigation, and improving farmers' access to credit.

This study focuses on only the credit module scheme of the NRGP. In the bid to ensure uniformity in comparisons of outputs, the study further focused on only maize farmers within the data set. The general impressions of farmers about NRGP credit interventions were largely positive, albeit the lower impressions of the female and youthful respondents. h. The youth expressed the most negative impressions because they felt ignored during the Programme implementation. The attitude of farmers to NRGP was generally poor in areas where farmers expected programme-facilitated credit support but never received one from the participating banks. Under the credit intervention model, NRGP instituted a partnership between input providers and participatory banks. Participatory banks gave interested and eligible farmers t a credit coupon to receive input to the limit of the credit coupons from the input providers. The maximum credit given to each farmer under this intervention was 530 Ghana cedis equivalent value of inputs. Farmers paid back the credit after harvesting.

## 2 Methodology

### 2.1 Study area

The population for this study comprises farmers within the operational areas of the Northern Rural Growth Program. These are farmers in the Northern, Upper East and Upper West Regions including seven adjoining districts of the Brong-Ahafo Region. The total number of maize farmers on the NRGP credit model is 1200.

### 2.2 Data set and sampling

The data type used for this study is cross-sectional, focusing on only credit-constrained maize farmers (maize farmers with daily income less than $1.9 per each member of the household and dependants) to allow for easy comparisons of the outcome variable; different variables are observed at a given period. These variables(age, gender, sex, number of dependence, experiences, level of education and training) are used to ascertain their homogeneity among participants and their collective impact on the outcome variable with or without the credit intervention.

The outcome variable, farm output per acre, is already given in the data set and the ages of farmers and the sex of each farmer. Therefore, there were follow-up phone calls to ascertain the status of the number of dependencies of each participant, levels of education, and experiences of each farmer measured in years. The inclusion of these variables for the study are their anticipated effects on the outcome variable and their inclusion onto the dataset will make the model more viable.

The first step in calculating the required sample size is to propose the expected outcome values for the counterfactual and then an expectation about the mean and the standard deviation of the outcome variable in the absence of the program. These values were approximated for the purpose of this study using pre-intervention averages of the outcome variable from survey information obtained from the Ministry of Food and Agriculture in Ghana. The mean value of output for the control group is proposed to be 3150kg and the mean value of output for treatment group is 3850kg whilst the standard deviation is proposed to be 1420kg. Secondly, the study proposed the treatment group’s expected outcome values. To determine these values, there is a need to dig into the literature to find similar program effects that have been estimated before. In this case the study used pre-intervention averages from Alliance for Financial Inclusion in their 2018 report on Agricultural Finance Intervention in Ghana, where it is assumed that, on average, the credit program increases the total farm output by 1420kg per hector (see appendix 1 for the sample computed using a code in Stata software). This study considered an 80% power in the sample calculation and therefore has an 80% chance of having significant results.

The dataset contains 1200 observations of credit-constrained maize farmers and has age, sex, education, output, number of dependents, marital status, and access to training as its variables. Additionally, the treatment variable takes a value of 1 if a farmer benefited from the NRGP credit intervention program (treatment group) and 0 otherwise (control group). There are 540 treated farmers and 660 in the control group. The study used a total sample size of 130 farmers, 65 farmers as treatment group and 65 farmers as control group. Farmers interviewed consented to give information for academic purpose and specifically for publication. Before the interview was conducted, the Kwame Nkrumah University of Science and Technology ethics committee approved the interview guide in accordance with the University research ethics.

### 2.3 Model specification

There are several impact evaluation techniques/models (Abdia et al., 2017), and they can be categorized into experimental techniques, quasi-experimental techniques, and non-experimental techniques (Carter, 1989). This study is a quasi-experimental research design; like experimental designs, the program is an intervention in which a treatment has been evaluated to how well it achieves its objectives. This study differs from experimental designs because it lacks random assignment to treatment and control groups (Abdia et al., 2017). However, assignment to treatment versus control is through self-selection or administrator selection or both of these.

The strongest quasi-experimental designs for causal inferences are regression discontinuity designs, instrumental variable designs, matching and propensity score designs, and comparative interrupted time series designs (Carter, 1989). This paper explored the propensity score designs in evaluating the impact of credit access on-farm productivity in Ghana using data from the NRGP.

### 2.4 Matching and propensity score designs

According to Blundell and Smith (1989), the work of Heckman, his co-authors and others points out that matching estimators perform well when;

1. The same set of questions are used for the treatment group and control group
2. Both the treated and control groups reside in the same geographical area
3. The data contains sets of variables (for this study, the variables used are age, education, number of dependence and farm experience) relevant to modelling the program participation decisions.

The data satisfies the above conditions and is therefore justified using the matching and propensity score approach. The matching method works by re-weighing the control group sample to provide a valid estimate of the counterfactual of interest (Abdia et al., 2017). After the re-weighing scheme, treatment and control units look the same observables. Under the matching assumption, the only remaining difference between the two groups is program participation. So, any difference in outcome between the treatment and control groups could be attributed to the treatment effect, provided no further systematic difference between these two groups other than those observables are established. The propensity score is formally defined in Eq. (1) as the conditional probability of receiving the treatment given the set of covariates X:

(1) PS=Pr(Z=1/X)

Matching and Propensity scores designs usually investigate Average Treatment Effect (ATE) (Abdia et al., 2017) and this is mathematically expressed in Eqs. (2) and (3).

(2) $$\left(ATE=\sum \left(Y_{i}\left(1\right)\right)-\sum \left(Y_{i}\left(0\right)\right)\right)$$

Where *Yi(1)* is the output of a farmer who had access to credit and *Yi(0)* is the output of a farmer who had no access to credit, and a causal effect is identified if;

(a) The farmer output is statistically independent of the credit access given the set of observed confounders (age, gender, size of dependence, education, and years of experience).
(b) The credit access has a probability strictly between zero and one (positivity assumption). Because the outputs of farmers who had access to credit and those who had no access to credit are observed, ATE is identified because it can be expressed in terms of observable quantities:

(3) $$ATE=\sum x\left(E\left(Y_{i}/Z=1;\;X\right)-\sum x\left(E\right)\left(Y_{i}/Zi=0;\;X\right)\right)$$

Where ∑x [E(Yi/Z=1; X)], represents the sum of the expected values of the output of the farmers with access to credit given a set of confounders (X) and ∑x[E(Yi/Z=0;X)], is the sum of the expected output of farmers with no access to credit given a set of confounders (X). The ATE then investigates the differences between these outputs, and a positive value means the credit access led to an increase in output. Also, a negative value shows that the credit access led to a decrease in output whilst zero (0) ATE indicates the credit access had no impact on output.

## 3 Results and discussion results

### 3.1 Data description

The variables used in this study are credit access, ages of participants, gender of each participant, number of dependants on each participant, experiences of each participant in farming activities measured in years, educational level of each participant and a total output of each participant measured in kilograms of farm output per acre. Although these variables are carefully selected to reduce bias, according to Abu and Haruna (2017), the choice of variables to be included in the PSM should be influenced by their relationship to the treatment and the outcome variables. Therefore, these variables are used for the PSM analysis because they are unrelated to credit access but related to farmer productivity.

The treatment variable is created as *crd* and takes a value of 1 if a farmer benefited from the credit program (treatment group) and 0 otherwise (control group). Dummies are created for sex, generated as a *gender* which takes the value of 1 if a farmer is a male and 0 otherwise and secondary education as 1 if a farmer has attained education to senior high school level and 0 otherwise and generated as *edu.* The variables considered for the PSM (age, sex, level of education, number of dependants and level of farming experience) were the same variables on the credit application forms. These variables influenced the credit administrators in deciding which credit application to be accepted and which one to be denied. These variables were analyzed using descriptive statistics such as mean, percentage and frequency distributions. In limiting the analysis to the data of the NRGP, results from this analysis are assumed to be the factors that determine farmers' credit access in Ghana. All the predictor variables were statistically significant and therefore are considered the determinants of credit access in Ghana.

The administrators of the credit model of the program asserted that the following reasons were the determinants of acceptance or rejection of the credit applications.

a. Incomplete credit application forms.
b. Inconsistent information provided by the applicants
c. Timing of the application
d. Non-guaranteed applicants

Participants' experiences and output are quantitative variables and therefore assumed their absolute values in the analysis. It is expected that credit interventions program will increase farm output in the short run. The selected outcome variable is the total outputs in kilograms per hector. Table 1 below summarises the quantitative data used in the study. Table 2 and Table 4 compared a summary of each of the basic statistics for control and treated farmers. Table 4 reveals the rigourous nature of the matching method used as the differences in the statistics are negligible.

**Table 1:** Summary statistics.

Variables	Observations	Min	Max	Mean	STD. DEV
Age	130	20.00	87.00	48.20	16.71
No. of Dep.	130	1.00	12.00	4.97	2.13
Experience	130	2.00	12.00	6.35	2.03
Output in Kg	130	1850.00	4856.00	3351.20	678.39
Sex	130	0.00	1.00	0.52	0.50
Education	130	0.00	1.00	0.46	0.50

Source: authors construct 2021.

**Table 2:** Summary of the matched observations.

Categories	Frequency	Matched Participants	Percentages of Matched Participants	Unmatched Participants	Percentages of Unmatched Participants
Control Group	65	26	40%	39	60%
Treated Group	65	26	40%	39	60%

Source: authors construct 2021.

### 3.2 The matching and propensity score results

From Table 2, 40% of the participants in the treatment group were matched to the control group. This is where candidates are found within the caliper radius of 0.10∗ sigma (for most of the matching, we had a bias lower than 10%). Here is a randomized list of all the participants in the treatment group. The first farmer in the treatment group is selected. Next, all farmers in the control group with a lower propensity score than previously chosen were selected (According to Yongji et al. 2013, when some of the covariates are continuous, the choice of caliper at or near 0.2 will yield superior results and so this study used 0.1 times the standard deviation of the general propensity score). The nearest Mahalanobis metric defines the final control for the matching among these control candidates. Suppose there is no control candidate within the caliper (only if the control's propensity score is within a 0.10 radius (caliper)). In that case, the procedure will fail to find a perfect match for the treatment. Thus, in this method, it is possible that a farmer with credit intervention cannot be matched to a control farmer without credit. These calipers can avoid bad matches and the closest propensity score is used to define the final control. The procedure runs until each farmer in the treatment group has one control. Then the procedure is performed again to find the second control in the sample without the controls already selected. Twenty-six farmers in the treatment group had perfect matches in the control group within the caliper radius of 0.10. These matched participants are completely similar in terms of their underlining characteristics, and any difference that results from their average outputs will be because of the credit intervention.

Table 3 above is the Wald Chi-Square and Pr > Chi^2^ results from the optimal algorithm Mahalanobis matching. The Wald Chi-Square is the test statistic, and the Pr > Chi^2^ are the p-values for each of the predictor variables for the hypothesis test that an individual predictor's regression coefficient is zero given the rest of the predictors are in the model. The Wald Chi-Square test is the squared ratio of the value estimate to the standard error of the respective predictor. The probability that a particular Wald Chi-Square test is as extreme as, or more so, than what has been observed under the null hypothesis is given by Pr > Chi^2^. It can be observed that there is no evidence of multicollinearity since none of the predictor variables has a standard error larger than 2 and indicates that all our explanatory variables are statistically significant at 95% confidence level.

**Table 3:** Standardized coefficients (accessed credit).

Source	Value	Standard error	Wald Chi-Square	Pr > Chi^2^
Age	0.220	0.125	3.117	0.007
No. of Dependance	0.086	0.121	0.506	0.027
Exp-yrs	0.403	0.129	9.811	0.002
Output-kg	-0.669	0.145	21.198	<0.0001
Sex-0	0.000	0.000		
Sex-1	0.135	0.120	1.270	0. 001
Sec-Educ-0	0.000	0.000		
Sec-Educ-1	0.019	0.126	0.022	0. 032

Source: authors construct 2021.

**Table 4:** Comparison of the basic statistics of the 26 matched pair of farmers.

Variable	Mean of Treated Group	Mean of Control Group	Mean Differences
Age	50.54	47.96	2.58
No. of Dependence	5.38	5.23	0.15
Experience in Years	6.77	6.54	0.23
Sex	0.46	0.54	(0.08)
Education	0.54	0.43	0.12
OUTPUT (KG)	3308.96	3265.27	44.69

Source: authors construct 2021.

### 3.3 Covariate balancing and sensitivity

**Figure 1:**
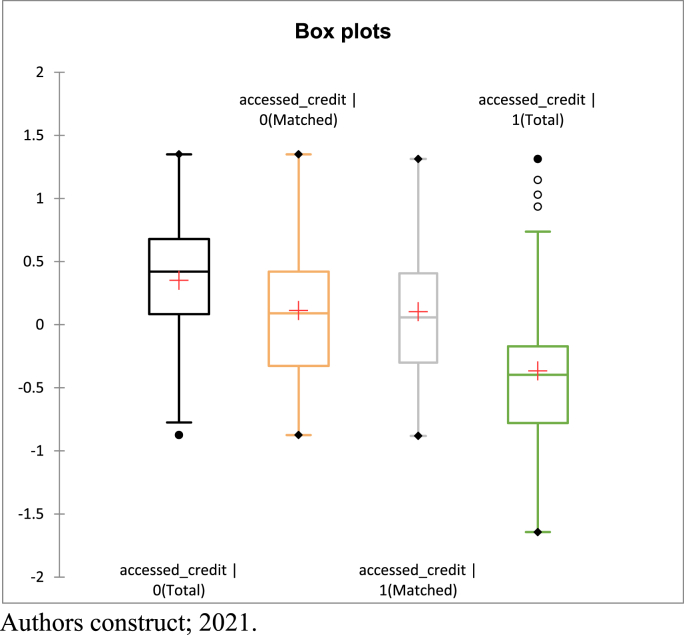
Covariate balancing.

The study provided results of covariate balancing after the propensity score estimation. When the covariate distribution does not vary over the treatment levels, the covariate is said to be balanced. If the matched sample box plots are the same over the treatment levels, the covariate is balanced in the matched sample. This is presented in Figure 1 (Box Plot). From the box plot, the matched sample is very similar. The medians and percentiles appear to be the same, although there may be some differences in the tails and the outliers. Still, the upper adjacent and lower adjacent values appear to be the same. Matching on the estimated propensity score appear to have balanced. Figure 1; Box Plots.

### 3.4 Average Treatment Effect (ate)

Given the p-score values in AppendixC, the Average Treatment Effect (ATE) estimation among the 52 farmers (26 farmers who had access to credit against their matched counterparts) is a simple difference of means between them. The most convenient way to estimate the difference in their means outputs is using the student t-test. The difference of means between the two groups is computed in Table 5.

**Table 5:** T-test for two paired samples/Two-tailed test.

Difference	-43.692
(Mean Value of Control Group)	3265.269
(Mean Value of Treated Group)	3308.962
p-value (Two-tailed)	0.041

The Welch-Satterthwaite formula approximates the number of degrees of freedom. Source; authors construct 2021.

95% confidence interval on the difference between the means [-296.879, 209.494].

The t-test results found the difference in the average output to be significant, t(44) = -25, p less than 0.05. This suggests that the credit intervention could be concluded to increase output by 44 kg of Maize.

## 4 Conclusion and recommendation

### 4.1 Conclusion

One of the roles of NRGP was to build the capacity of financial institutions on value chain financing, credit appraisal, cash flow analyses, financial management skills, credit delivery and administrations among others. Though this study focuses only on the credit intervention program of the NRGP, the program did achieved its intended purpose by improving farm productivity in Ghana. It could therefore be deduced that; credit interventions programs do have a positive impact on farm productivity in Ghana.

### 4.2 Recommendation

For agribusiness to succeed in Ghana, there is a strong need to increase credit to farmers who are credit constraint. Credit-constrained farmers could be easily identified by accessing credible farm data such as the agricultural census data from the Ministry of Food and Agriculture. Farmers differ in their needs for credit and therefore If the specific credit needs of farmers are not considered, the marginal effect of the credit on farm output may not be significant.

## Declarations

### Author contribution statement

Mutaka Mohammed Seidu: Conceived and designed the experiments; Performed the experiments; Analyzed and interpreted the data.

Mohammed Tanko: Contributed reagents, materials, analysis tools or data; Wrote the paper.

### Funding statement

This research did not receive any specific grant from funding agencies in the public, commercial, or not-for-profit sectors.

### Data availability statement

The authors do not have permission to share data.

### Declaration of interest’s statement

The authors declare no conflict of interest.

### Additional information

No additional information is available for this paper.

## Figures and Tables

**Figure:**
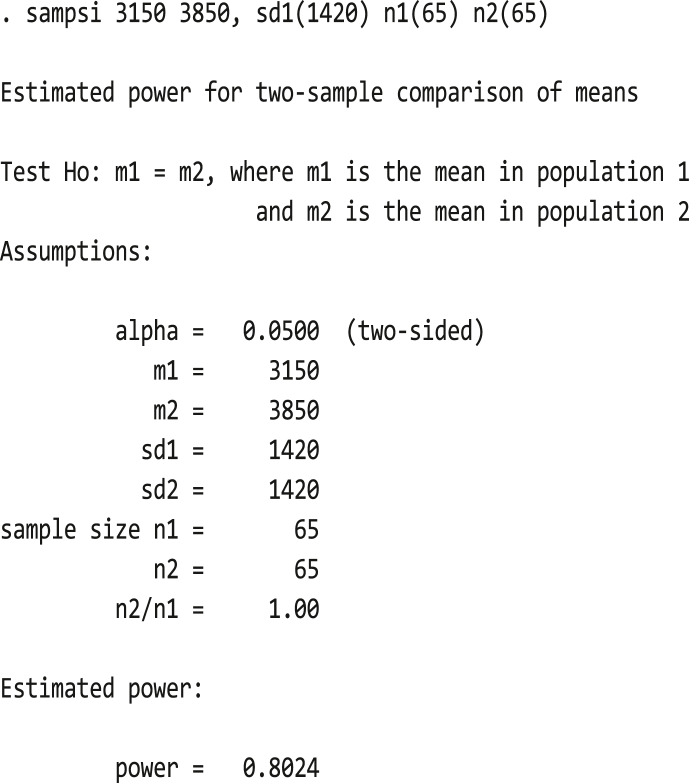


**Table:** 

Output of Treated Participants	Logit (Propensity score)	Output of Control Participants	Logit (Propensity score)	Distances
3560	0.560	2545	0.562	0.004
2265	1.039	2157	1.030	0.017
3105	0.064	2489	0.038	0.051
1950	1.111	2469	1.147	0.072
2984	0.083	2601	0.057	0.054
2990	-0.482	3850	-0.524	0.084
3856	-0.327	3973	-0.319	0.016
3658	0.096	2895	0.058	0.076
3865	-0.122	3658	-0.171	0.099
2985	0.373	2759	0.393	0.039
3799	-0.522	3568	-0.524	0.004
4525	-0.874	3745	-0.882	0.015
2968	0.212	2854	0.162	0.100
4856	-0.574	3745	-0.579	0.009
2506	-0.249	3875	-0.249	0.001
3899	-0.020	3985	0.014	0.069
3987	-0.775	3785	-0.779	0.008
3687	0.136	3987	0.158	0.043
2890	0.176	3852	0.171	0.009
3590	-0.231	4789	-0.233	0.004
3560	0.742	3121	0.737	0.010
2869	0.230	2900	0.197	0.066
2998	0.421	2987	0.412	0.017
3560	-0.452	3397	-0.433	0.039
2867	0.958	2961	0.935	0.046
2254	1.350	1950	1.313	0.073

**Table:** 

Observation	Propensity score	Lower bound 95%	Upper bound 95%	Logit (Propensity score)	Lower bound 95%	Upper bound 95%
Obs1	0.933	0.785	0.982	1.147	0.564	1.730
Obs2	0.113	0.042	0.269	-0.896	-1.358	-0.434
Obs3	0.200	0.090	0.389	-0.602	-1.007	-0.197
Obs4	0.092	0.033	0.230	-0.996	-1.466	-0.526
Obs5	0.730	0.490	0.884	0.432	-0.018	0.883
Obs6	0.829	0.630	0.932	0.684	0.230	1.139
Obs7	0.107	0.039	0.264	-0.919	-1.394	-0.445
Obs8	0.123	0.044	0.302	-0.852	-1.340	-0.364
Obs9	0.784	0.605	0.896	0.560	0.186	0.935
Obs10	0.725	0.541	0.856	0.422	0.071	0.773
Obs11	0.916	0.755	0.975	1.039	0.489	1.588
Obs12	0.112	0.038	0.287	-0.898	-1.403	-0.394
Obs13	0.537	0.213	0.832	0.064	-0.569	0.696
Obs14	0.030	0.007	0.122	-1.512	-2.166	-0.857
Obs15	0.170	0.063	0.385	-0.688	-1.172	-0.204
Obs16	0.761	0.537	0.898	0.503	0.064	0.942
Obs17	0.184	0.075	0.385	-0.646	-1.089	-0.203
Obs18	0.106	0.034	0.283	-0.926	-1.448	-0.404
Obs19	0.485	0.233	0.744	-0.026	-0.516	0.464
Obs20	0.221	0.090	0.449	-0.546	-1.004	-0.088
Obs21	0.812	0.640	0.913	0.634	0.250	1.019
Obs22	0.372	0.190	0.599	-0.228	-0.629	0.174
Obs23	0.170	0.050	0.444	-0.688	-1.277	-0.098
Obs24	0.928	0.777	0.979	1.111	0.543	1.679
Obs25	0.204	0.067	0.476	-0.593	-1.144	-0.041
Obs26	0.678	0.440	0.850	0.324	-0.105	0.753
Obs27	0.877	0.699	0.956	0.852	0.366	1.338
Obs28	0.066	0.021	0.194	-1.149	-1.678	-0.620
Obs29	0.230	0.105	0.432	-0.524	-0.929	-0.119
Obs30	0.165	0.069	0.345	-0.703	-1.129	-0.278
Obs31	0.548	0.284	0.787	0.083	-0.402	0.568
Obs32	0.695	0.491	0.843	0.357	-0.016	0.731
Obs33	0.043	0.010	0.164	-1.348	-1.990	-0.707
Obs34	0.022	0.004	0.104	-1.643	-2.350	-0.937
Obs35	0.799	0.580	0.920	0.600	0.140	1.060
Obs36	0.360	0.136	0.668	-0.249	-0.802	0.304
Obs37	0.116	0.030	0.361	-0.882	-1.515	-0.248
Obs38	0.777	0.459	0.935	0.542	-0.071	1.155
Obs39	0.369	0.206	0.568	-0.233	-0.586	0.119
Obs40	0.248	0.096	0.505	-0.482	-0.973	0.009
Obs41	0.100	0.032	0.273	-0.955	-1.485	-0.425
Obs42	0.392	0.206	0.617	-0.190	-0.587	0.207
Obs43	0.106	0.029	0.321	-0.925	-1.525	-0.326
Obs44	0.712	0.512	0.853	0.393	0.021	0.765
Obs45	0.209	0.044	0.602	-0.579	-1.337	0.180
Obs46	0.399	0.197	0.642	-0.178	-0.609	0.253
Obs47	0.721	0.510	0.865	0.412	0.018	0.807
Obs48	0.320	0.129	0.599	-0.327	-0.829	0.175
Obs49	0.763	0.522	0.905	0.507	0.038	0.977
Obs50	0.286	0.146	0.484	-0.398	-0.767	-0.029
Obs51	0.868	0.649	0.959	0.817	0.266	1.368
Obs52	0.175	0.076	0.354	-0.672	-1.083	-0.262
Obs53	0.555	0.299	0.785	0.096	-0.369	0.562
Obs54	0.107	0.034	0.288	-0.923	-1.453	-0.393
Obs55	0.430	0.249	0.632	-0.122	-0.479	0.235
Obs56	0.683	0.438	0.857	0.334	-0.109	0.777
Obs57	0.403	0.213	0.627	-0.171	-0.569	0.226
Obs58	0.886	0.703	0.962	0.890	0.375	1.405
Obs59	0.486	0.252	0.726	-0.024	-0.472	0.424
Obs60	0.703	0.500	0.848	0.373	0.000	0.747
Obs61	0.533	0.279	0.772	0.058	-0.412	0.529
Obs62	0.231	0.081	0.506	-0.522	-1.055	0.011
Obs63	0.801	0.562	0.926	0.604	0.109	1.098
Obs64	0.508	0.251	0.761	0.014	-0.475	0.503
Obs65	0.118	0.039	0.304	-0.874	-1.389	-0.359
Obs66	0.818	0.536	0.946	0.654	0.063	1.244
Obs67	0.324	0.131	0.604	-0.319	-0.821	0.183
Obs68	0.683	0.446	0.852	0.333	-0.095	0.761
Obs69	0.619	0.359	0.826	0.212	-0.252	0.676
Obs70	0.357	0.173	0.595	-0.256	-0.678	0.166
Obs71	0.590	0.386	0.767	0.158	-0.202	0.517
Obs72	0.366	0.203	0.566	-0.240	-0.594	0.115
Obs73	0.367	0.186	0.595	-0.237	-0.641	0.167
Obs74	0.817	0.589	0.933	0.650	0.156	1.144
Obs75	0.533	0.256	0.790	0.057	-0.463	0.576
Obs76	0.210	0.094	0.407	-0.574	-0.984	-0.164
Obs77	0.915	0.717	0.978	1.030	0.404	1.656
Obs78	0.312	0.145	0.550	-0.343	-0.772	0.087
Obs79	0.684	0.483	0.834	0.336	-0.030	0.701
Obs80	0.896	0.729	0.965	0.935	0.430	1.440
Obs81	0.735	0.429	0.911	0.443	-0.125	1.010
Obs82	0.882	0.642	0.969	0.872	0.254	1.491
Obs83	0.316	0.162	0.526	-0.335	-0.715	0.044
Obs84	0.827	0.583	0.942	0.678	0.145	1.211
Obs85	0.592	0.330	0.810	0.162	-0.307	0.630
Obs86	0.869	0.659	0.958	0.823	0.287	1.359
Obs87	0.874	0.693	0.955	0.841	0.354	1.328
Obs88	0.472	0.284	0.669	-0.049	-0.402	0.305
Obs89	0.954	0.790	0.991	1.313	0.575	2.052
Obs90	0.522	0.259	0.773	0.038	-0.457	0.533
Obs91	0.186	0.052	0.487	-0.642	-1.260	-0.023
Obs92	0.361	0.165	0.616	-0.249	-0.704	0.206
Obs93	0.488	0.286	0.695	-0.020	-0.398	0.358
Obs94	0.176	0.071	0.374	-0.671	-1.119	-0.223
Obs95	0.144	0.055	0.325	-0.775	-1.232	-0.318
Obs96	0.350	0.157	0.608	-0.269	-0.729	0.191
Obs97	0.578	0.336	0.787	0.136	-0.296	0.568
Obs98	0.658	0.437	0.827	0.284	-0.110	0.678
Obs99	0.461	0.260	0.676	-0.067	-0.453	0.319
Obs100	0.611	0.234	0.890	0.197	-0.515	0.909
Obs101	0.358	0.170	0.602	-0.254	-0.688	0.180
Obs102	0.866	0.676	0.952	0.809	0.319	1.299
Obs103	0.856	0.669	0.946	0.774	0.306	1.243
Obs104	0.600	0.324	0.824	0.176	-0.319	0.670
Obs105	0.143	0.057	0.313	-0.779	-1.216	-0.342
Obs106	0.370	0.119	0.718	-0.231	-0.869	0.406
Obs107	0.847	0.603	0.953	0.742	0.182	1.303
Obs108	0.687	0.460	0.849	0.341	-0.069	0.751
Obs109	0.081	0.024	0.238	-1.057	-1.610	-0.505
Obs110	0.641	0.426	0.812	0.252	-0.130	0.634
Obs111	0.356	0.175	0.590	-0.258	-0.674	0.158
Obs112	0.828	0.626	0.933	0.683	0.223	1.143
Obs113	0.209	0.088	0.418	-0.579	-1.015	-0.143
Obs114	0.629	0.365	0.834	0.230	-0.240	0.700
Obs115	0.093	0.022	0.320	-0.992	-1.655	-0.328
Obs116	0.270	0.101	0.549	-0.433	-0.951	0.086
Obs117	0.785	0.504	0.929	0.562	0.006	1.118
Obs118	0.845	0.645	0.943	0.737	0.259	1.215
Obs119	0.597	0.310	0.830	0.171	-0.348	0.690
Obs120	0.792	0.619	0.899	0.580	0.211	0.950
Obs121	0.230	0.078	0.515	-0.524	-1.073	0.025
Obs122	0.759	0.445	0.925	0.497	-0.097	1.091
Obs123	0.757	0.495	0.908	0.493	-0.008	0.994
Obs124	0.725	0.523	0.864	0.421	0.040	0.801
Obs125	0.261	0.104	0.519	-0.452	-0.937	0.033
Obs126	0.313	0.141	0.560	-0.340	-0.785	0.104
Obs127	0.681	0.408	0.868	0.328	-0.162	0.819
Obs128	0.901	0.738	0.967	0.958	0.449	1.466
Obs129	0.957	0.812	0.991	1.350	0.635	2.064
Obs130	0.886	0.643	0.971	0.892	0.256	1.529

**Table:** 

Treatment	Logit (Propensity score)	Control	Logit (Propensity score)	Distances
Obs9	0.560	Obs117	0.562	0.004
Obs11	1.039	Obs77	1.030	0.017
Obs13	0.064	Obs90	0.038	0.051
Obs24	1.111	Obs1	1.147	0.072
Obs31	0.083	Obs75	0.057	0.054
Obs40	-0.482	Obs121	-0.524	0.084
Obs48	-0.327	Obs67	-0.319	0.016
Obs53	0.096	Obs61	0.058	0.076
Obs55	-0.122	Obs57	-0.171	0.099
Obs60	0.373	Obs44	0.393	0.039
Obs62	-0.522	Obs29	-0.524	0.004
Obs65	-0.874	Obs37	-0.882	0.015
Obs69	0.212	Obs85	0.162	0.100
Obs76	-0.574	Obs45	-0.579	0.009
Obs92	-0.249	Obs36	-0.249	0.001
Obs93	-0.020	Obs64	0.014	0.069
Obs95	-0.775	Obs105	-0.779	0.008
Obs97	0.136	Obs71	0.158	0.043
Obs104	0.176	Obs119	0.171	0.009
Obs106	-0.231	Obs39	-0.233	0.004
Obs107	0.742	Obs118	0.737	0.010
Obs114	0.230	Obs100	0.197	0.066
Obs124	0.421	Obs47	0.412	0.017
Obs125	-0.452	Obs116	-0.433	0.039
Obs128	0.958	Obs80	0.935	0.046
Obs129	1.350	Obs89	1.313	0.073

## Appendix A Stata code and results for power calculation and sample size for each treatment and control group.

The above code generated the study's required power, n1 is the sample size for the treatment group, and n2 is the sample size required for the control group.

## Appendix B Farm Outputs of Matched Observations

The outputs associated with each pair of matched farmers are exhibited with their propensity score. These observations will aid in further determination of the treatment effect.

## References

[bib1] Abdallah A. (2016). Agricultural credit and technical efficiency in Ghana: is there a nexus?. Agric. Finance Rev..

[bib2] Abdallah A. (2016). Does credit market inefficiency affect technology adoption? Evidence from Sub-Saharan Africa. Agric. Finance Rev..

[bib49] Abdia Y., Kulasekera K.B., Datta S., Boakye M., Kong M. (2017). Propensity scores based methods for estimating average treatment effect and average treatment effect among treated: a comparative study. Biometrical Journal.

[bib3] Abdulai A., Binder C.R. (2006). Slash-and-burn cultivation practice and agricultural input demand and output supply. Environ. Dev. Econ..

[bib4] Abdulai A., Owusu V., Goetz R. (2008). http://mpra.ub.uni-muenchen.de/37046/%5Cnhttps://mpra.ub.unimuenchen.de/37046/1/MPRA_paper_37046.pdf.

[bib5] Abebaw D., Haile M.G. (2013). The impact of cooperatives on agricultural technology adoption: empirical evidence from Ethiopia. Food Pol..

[bib6] Abu B.M., Haruna I. (2017). Financial inclusion and agricultural commercialization in Ghana: an empirical investigation. Agric. Finance Rev..

[bib9] Akram W., Hussaun Z., Sail M.H., Hussain J. (2008). Agricultural credit-constraineds and borrowing behavior of farmers in Rural Punjab. Eur. J. Sci. Res..

[bib10] Akudugu M. (2014). Estimating the effects of formal and informal credit on farm household welfare: a hierarchical competitive welfare model approach. J. Dev. Agric. Econ..

[bib11] Akudugu M. (2016). Agricultural productivity, credit and farm size nexus in Africa: a case study of Ghana. Agric. Finance Rev..

[bib12] Amanullah Ghulam Rasool, Lakhan Siraj, Ahmed Channa, Magsi Habibullah, Ahmed Koondher Mansoor, Wang Jing, Ahmed Channa Naseer (2020). Credit-constrained and rural farmers’ welfare in agrarian economy. Heliyon.

[bib13] Awotide B.A., Abdoulaye T., Alene A., Manyong V.M. (2015).

[bib14] Ayamga M., Dzanku F. (2013). The land rights and farm investment Ghana: the missing link in the operationalization of tenure security". Invited Paper Presented at the 4th International Conference of the African Association of Agricultural Economists.

[bib15] Bank of Ghana Report (2018).

[bib16] Binswanger H.P., Khandker S.R. (1995). The impact of formal finance on the rural economy of India. J. Dev. Stud..

[bib17] Blundell R.W., Smith R.J. (1989). Estimation in a class of simultaneous equation limited dependent variable models. Rev. Econ. Stud..

[bib18] Carter M.R. (1989). The impact of credit on peasant productivity and differentiation in Nicaragua. J. Dev. Econ..

[bib19] Dadson A.-V., Ramatu M., Al-Hassan D.B.S., Irene E. (2014). “Agricultural credit rationing in Ghnan: what do formal lenders look for?. Agric. Finance Rev..

[bib20] Dehejia R.H., Wahba S. (2002). Propensity Score-Matching Methods for Non-experimental Causal Studies. Review of Economics and Statistics.

[bib21] Di Falco S., Bulte E. (2011). A dark side of social capital? Kinship, consumption, and savings. J. Dev. Stud..

[bib22] Di Falco S., Veronesi M., Yesuf M. (2010).

[bib23] Doss C.R., Morris M.L. (2001). How does gender affact head option fagricultural innovations? The case of improved maize technology in Ghana. Agric. Econ..

[bib24] El-Shater T., Yigezu Y.A., Mugera A., Piggin C., Haddad A., Khalil Y., Loss S., Aw-Hassan A. (2016). Does zero tillage improve the livelihoods of smallholder cropping farmers?. J. Agric. Econ..

[bib25] FAPDA (2015). Socio-economic Context and Role of Agriculture, Country Fact Sheet on Food and Agriculture Policy Trends, Ghana. Food and Agriculture Organization of the United Nations.

[bib26] Foltz J.D. (2004). Credit Market Access and Profitability in Tunisian Agriculture. Agric. Econ..

[bib27] Foltz J.D. (2004). Credit market access and profitability in tunusian agriculture. Agric. Econ..

[bib28] Feder G., Lau L.J., Lin J.Y., Luo X. (1990). The relationship between credit and productivity in Chinese agriculture: a microeconomic model of disequilibrium. Am. J. Agric. Econ..

[bib29] Fletschner D., Guirkinger C., Boucher S. (2010). Risk, credit-constraineds, and financial efficiency in Peruvian agriculture. J. Dev. Stud..

[bib30] Fuglie K.O., Bosch D.J. (1995). Of soil implications nitrogen testing analysis. Am. J. Agric. Econ..

[bib31] Giang T.T., Wang G., Chien N.D. (2015). Impact of credit on poor household's income: evidence from rural areas of Vietnam. Journal of Finance and Economics.

[bib32] Ghana Statistical Service (2016).

[bib33] Guirkinger C., Boucher S.R. (2008). Credit-constraineds and productivity in Peruvian agriculture. Agric. Econ..

[bib34] Hammamet, Tunisia, PeruvianVol. 4 No. 9, pp. 1-22.

[bib35] Barrett C.B. (2001). Does food aid stabilize food availability?. Econ. Dev. Cult. Change.

[bib36] Hananu B., Abdallah A., Zakaria H. (2015). Factors influencing agricultural credit demand in Northern Ghana. Afr. J. Agric. Res..

[bib37] Iddrisu A., Gershon I., Ansah K., Nkegbe P.K. (2018). Effect of input credit on smallholder farmers' output and income evidence from Northern Ghana input credit. Agric. Finance Rev..

[bib38] Imbens G.W., Wooldridge J.M. (2009). Recent developments in the econometrics of program evaluation. J. Econ. Lit..

[bib39] Johnson M., Jimah K., Taabazuing J., Tenga A., Abokyi E., Nasser G., Owusu V. (2011).

[bib40] Kassie M., Zikhali P., Pender J., Köhlin G. (2008). Organic Farming Technologies and Agricultural Productivity: the Case of Semi-arid Ethiopia. Working papers in Economics, January, Göteborg.

[bib41] Kochar A. (1995). Explaining household vulnerability to idiosyncratic income shocks. Am. Econ. Rev..

[bib42] Martey E., Wiredu A.N., Etwire P.M. (2015).

[bib43] Minten B. (2002). Returns to social network capital among traders. Oxf. Econ. Pap..

[bib44] MoFA (2013). Agriculture in Ghana: facts and figures. Ministry of Food and Agriculture, Statistics, Research and Information Directorate (SRID), Accra.

[bib45] Mugumaarhahama Y. (2021).

[bib46] Natarajan N., Brickell K. (2021).

[bib47] Sekyi S., Abu B.M., Nkegbe P.K. (2017). Farm credit access, credit-constrained and productivity in Ghana: empirical evidence from Northern Savanna ecological zone. Agricultural Finance Review.

[bib48] Yongji W., Hongwei C., Chanjuam L., Zhiwei J., Ling W., Jiugang S., Jielai X. (2013). Optimal caliper width for propensity score matching of three treatement groups; A Monte Carlo study. PLoS One.

